# Supplementary material for: Cryo-EM structures define ubiquinone-10 binding to mitochondrial complex I and conformational transitions accompanying Q-site occupancy
Source: Nat Commun. 2022 May 19;13:2758. doi: 10.1038/s41467-022-30506-1 (PMC9120487; doi:10.1038/s41467-022-30506-1)
Supplement: Supplementary file 1 — Supplementary Information [file 41467_2022_30506_MOESM1_ESM.pdf]

Supplementary Information

Cryo-EM structures define ubiquinone-10 binding to mitochondrial complex I and conformational transitions accompanying Q-site occupancy

Injae Chung, John J. Wright[¶], Hannah R. Bridges[¶], Bozhidar S. Ivanov[¶], Olivier Biner, Caroline S. Pereira, Guilherme M. Arantes, Judy Hirst*

[¶]These authors contributed equally.

*Address correspondence to:

Judy Hirst, MRC Mitochondrial Biology Unit, University of Cambridge, The Keith Peters Building, Cambridge Biomedical Campus, Hills Road, Cambridge, CB2 0XY, UK

Tel: +44 1223 252810, E-mail: jh@mrc-mbu.cam.ac.uk

This file includes:

Supplementary Table 1

Supplementary Figs. 1 to 10

Supplementary Notes 1 to 2

References

Supplementary Information

Contents:

Supplementary Table 1	Map-to-map correlations and RMSD values between maps and models determined here and available in the protein and electron microscopy data banks.
Supplementary Fig. 1	Purification and biochemical characterisation of *Bos taurus* complex I reconstituted into nanodiscs (CxI-NDs).
Supplementary Fig. 2	Cryo-EM data processing – global classification.
Supplementary Fig. 3	Cryo-EM data processing – local classification.
Supplementary Fig. 4	Local resolution maps, Mollweide projections, 3DFSC plots, and Fourier shell correlation curves for the five CxI-ND states.
Supplementary Fig. 5	Densities in the CxI-ND maps for key elements that differ between the states and representative densities for water molecules.
Supplementary Fig. 6	Structural features of state 3 of bovine complex I.
Supplementary Fig. 7	Structural features of complex I-reconstituted nanodiscs.
Supplementary Fig. 8	Free energy profiles for additional structural properties obtained from metadynamics simulations.
Supplementary Fig. 9	Features of the Coulomb potential density for the ligand in the deactive-ligand map.
Supplementary Fig. 10	Comparisons between cryo-EM and X-ray crystal structures of ligands bound in the Q-binding site of complex I.
Supplementary Note 1	Biochemical relevance of the state 3 structure.
Supplementary Note 2	Minor differences between the structures of nanodisc-reconstituted and DDM-solubilised complex I.

Supplementary Table 1: Map-to-map correlations and RMSD values between maps and models determined here and available in the protein and electron microscopy data banks.

a	*B. taurus* (DDM)			*M. musculus* (DDM)	
	Active[1] EMD-14127	Deactive[2] EMD-3731	State 3[3] EMD-4041	Active[4] EMD-11377	Deactive[5] EMD-11810
Active-Q_{10}	0.97	0.93	0.93	0.97	0.95
Active-apo	0.97	0.93	0.93	0.97	0.95
Deactive-ligand	0.84	0.98	0.97	0.88	0.97
Deactive-apo	0.85	0.99	0.97	0.89	0.97
State 3	0.81	0.94	0.97	0.86	0.93

b	*O. aries*	*B. taurus*					*M. musculus*	
	Native[6]	Active		Deactive		State 3	Active[4]	Deactive[5]
		Q_{10}	Apo	Ligand	Apo		6ZR2 EMD-11377	7AK5 EMD-11810
Map-to-map	Closed EMD-11256	0.93	0.93	0.83	0.85	0.82	0.90	0.84
	Open1 EMD-11257	0.82	0.81	0.97	0.96	0.89	0.80	0.89
	Open2 EMD-11258	0.82	0.81	0.92	0.93	0.88	0.81	0.88
	Open3 EMD-11259	0.83	0.82	0.91	0.91	0.93	0.82	0.87
RMSD	Closed 6ZKO	0.81	0.81	2.35	2.32	3.98	1.19	2.14
	Open1 6ZKP	2.16	2.14	0.57	0.59	2.53	2.78	1.47
	Open2 6ZKQ	2.36	2.34	1.29	1.29	3.44	2.92	1.75
	Open3 6ZKR	3.21	3.18	1.39	1.42	1.33	3.87	2.14

Map-to-map correlation values were calculated using the *Fit in Map* tool in UCSF ChimeraX[7]. RMSD calculations were performed using the *Align* command in PyMOL[8]. Where relevant, PDB and EMDB codes are given as four-letter and five-digit codes, respectively. **a)** Comparisons of the maps determined here and those reported previously by our group for active, deactive, and state 3 structures of mammalian complex I. All the maps were low-pass filtered to the lowest resolution map in the table (published state 3 map at 5.6 Å resolution[3]), and the maps determined here were fitted into the published maps to calculate map-to-map correlation values. For comparisons, cells are shaded in colour scales from blue (best fit) to white (50th percentile) to red (worst fit). **b)** Comparisons of the maps and models determined here and those available in the PDB and EMDB. All the maps were low-pass filtered to the lowest resolution map in the table (published ovine closed map at 3.8 Å resolution[6]), and the ovine maps were fitted into the maps determined here to calculate map-to-map correlation values. For comparisons, cells are shaded in colour scales from blue (best fit) to white (50th percentile) to red (worst fit).

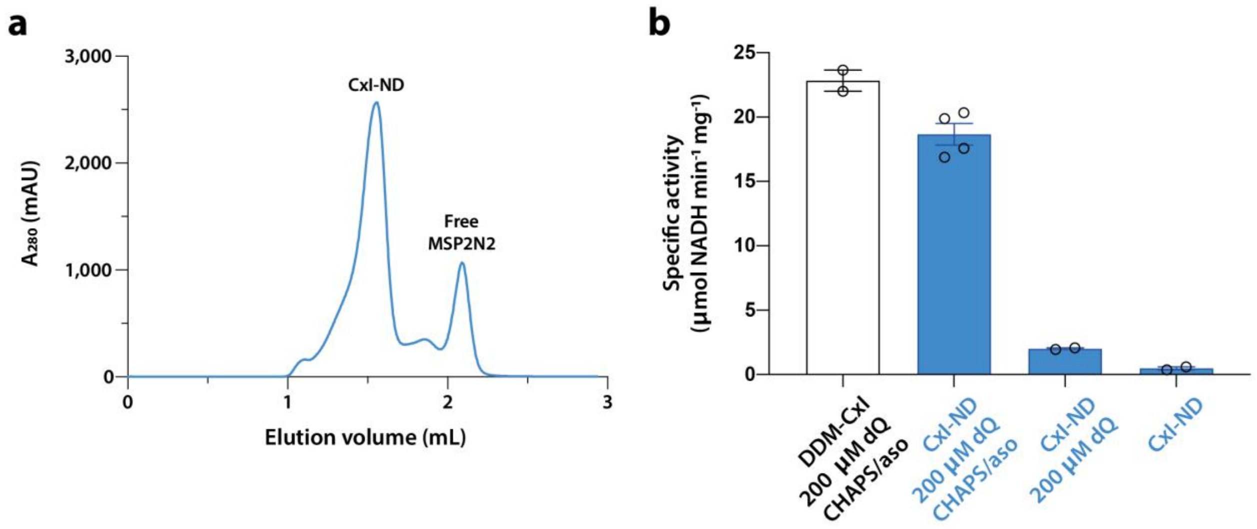

Supplementary Fig. 1: Purification and biochemical characterisation of *Bos taurus* complex I reconstituted into nanodiscs (CxI-NDs). a) Elution of CxI-NDs from the Superose 6 increase 5/150 size-exclusion column in the final step of the preparation (see Methods for details). The CxI-NDs elute at ~1.6 mL, the same as the DDM-bound enzyme. **b)** Specific NADH:dQ activity data on CxI-DDM and CxI-NDs show that the complex I in CxI-NDs is highly catalytically competent, but catalysis is limited by substrate access. The reference activity (DDM-CxI, before reconstitution) is conserved in CxI-NDs following addition of CHAPS (to dissociate them) and asolectin (to create a lipidic phase). Without CHAPS/asolectin the activity was much lower, so either dQ does not exchange effectively in and out of the nanodisc, its mobility is restricted, or the MSPs prevent it from entering the active site. The low observed rate of NADH oxidation, which is further decreased in the absence of dQ, may either result from dQ reduction by complex I, or dQ-mediated reoxidation of the $Q_{10}H_2$ generated by complex I. The data reported were recorded on the sample analysed by cryo-EM (n = 2 or 4 technical replicates), and presented as mean values ± SEM; note that inhibitor-insensitive background rates were not recorded in some cases due to lack of sample availability, and therefore all the data in the figure are reported without background subtraction to enable their comparison. 200 µM NADH was added in all cases (see Methods for experimental details).

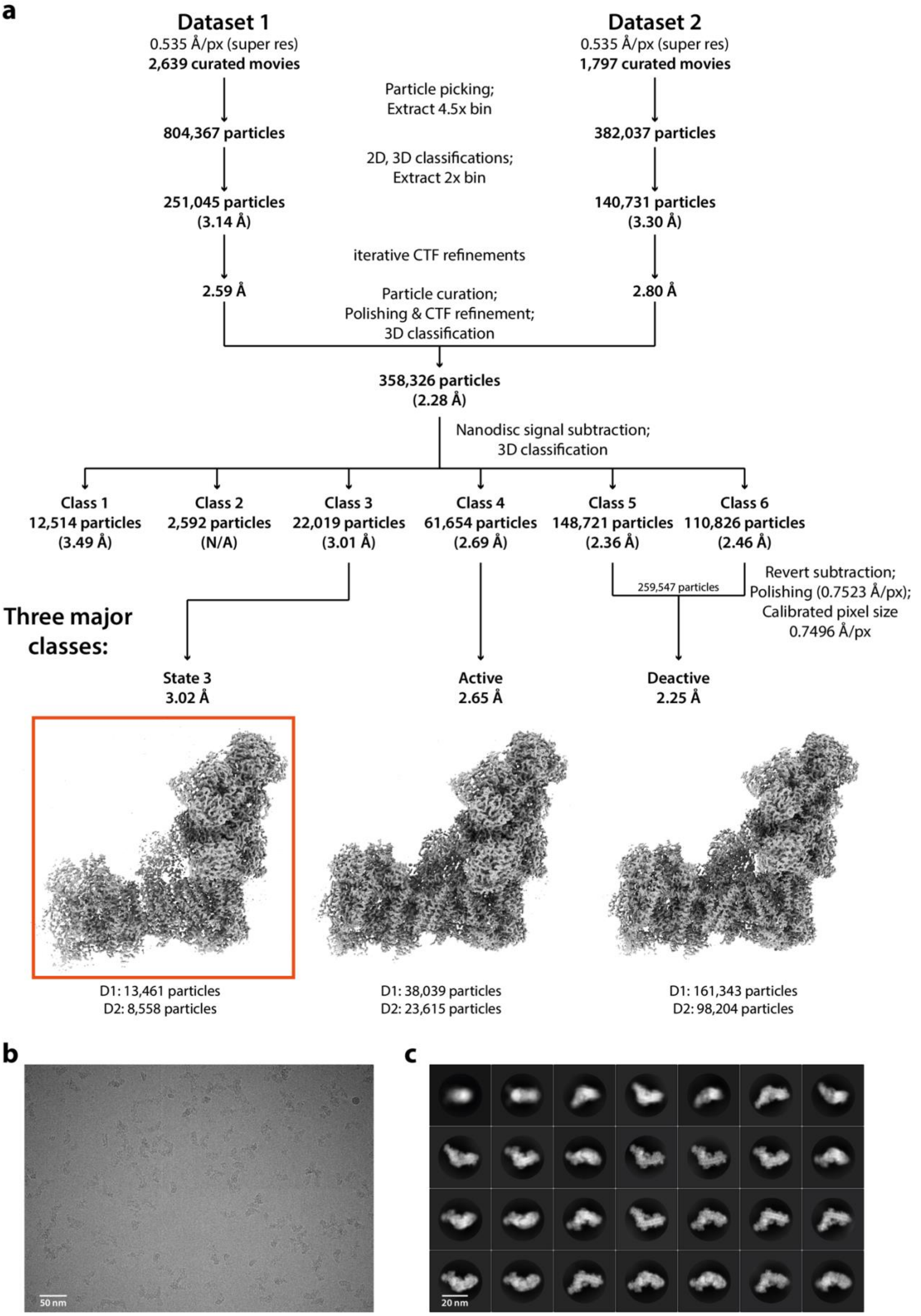
a
Dataset 1
0.535 Å/px (super res)
2,639 curated movies
Dataset 2
0.535 Å/px (super res)
1,797 curated movies
Particle picking;
Extract 4.5x bin
804,367 particles
382,037 particles
2D, 3D classifications;
Extract 2x bin
251,045 particles
(3.14 Å)
140,731 particles
(3.30 Å)
iterative CTF refinements
2.59 Å
2.80 Å
Particle curation;
Polishing & CTF refinement;
3D classification
358,326 particles
(2.28 Å)
Nanodisc signal subtraction;
3D classification
Class 1
12,514 particles
(3.49 Å)
Class 2
2,592 particles
(N/A)
Class 3
22,019 particles
(3.01 Å)
Class 4
61,654 particles
(2.69 Å)
Class 5
148,721 particles
(2.36 Å)
Class 6
110,826 particles
(2.46 Å)
259,547 particles
Revert subtraction;
Polishing (0.7523 Å/px);
Calibrated pixel size
0.7496 Å/px
Three major classes:
State 3
3.02 Å
Active
2.65 Å
Deactive
2.25 Å
D1: 13,461 particles
D2: 8,558 particles
D1: 38,039 particles
D2: 23,615 particles
D1: 161,343 particles
D2: 98,204 particles
b
50 nm
c
20 nm

Supplementary Fig. 2: Cryo-EM data processing – global classification. a) A flow chart of cryo-EM data processing up to the final global 3D classification leading to the three major classes – active, deactive and state 3. D1 and D2 denote Datasets 1 and 2, respectively. Where applicable, the red box denotes that this is the final map for the class. **b)** A representative raw micrograph from the data collections. A total of 6,780 micrographs were recorded. **c)** Representative 2D class averages from the data processing. The example view was selected following 2D classification of the final 3D refined particles to show classes of particles in different orientations.

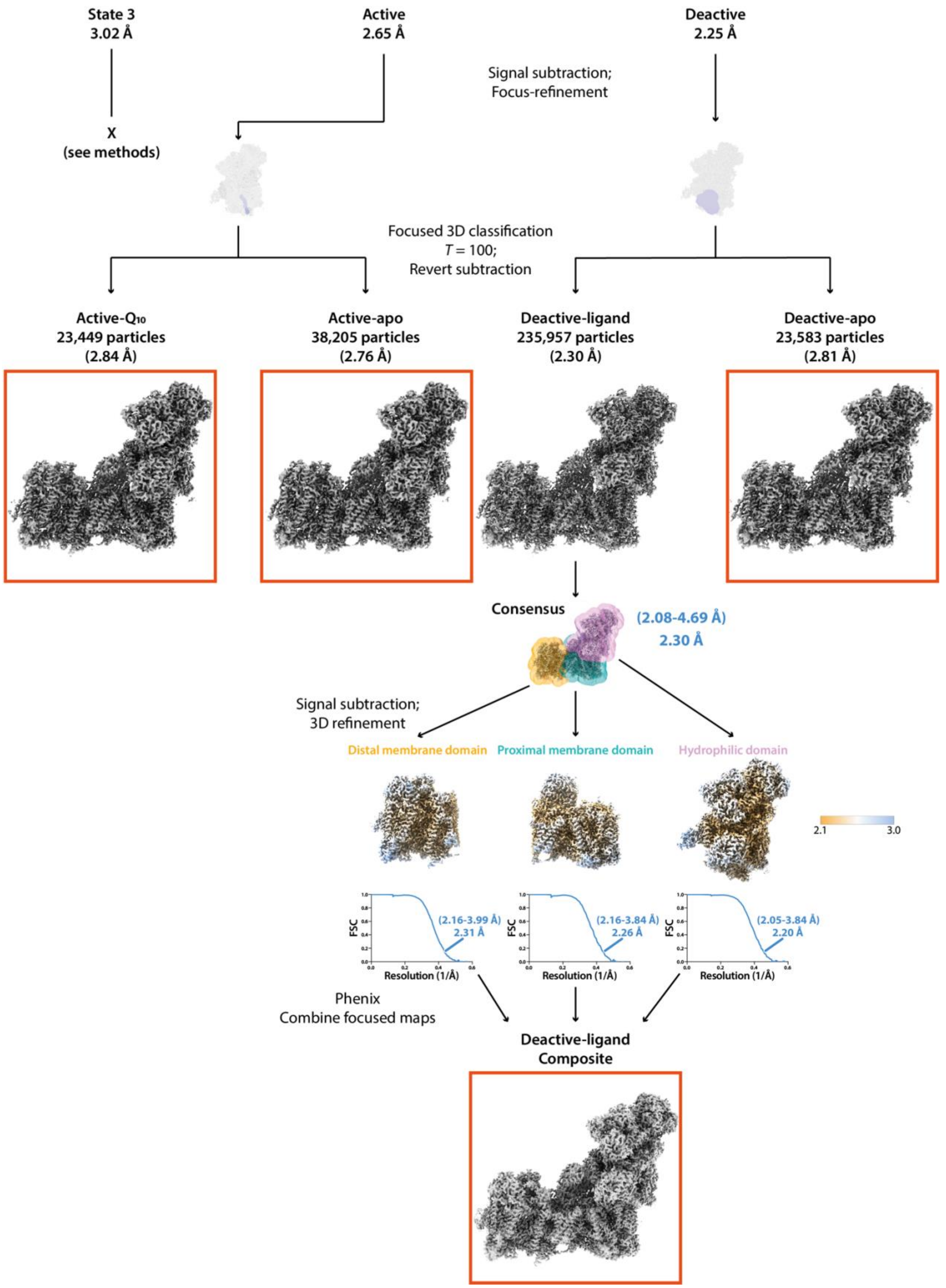
State 3
3.02 Å
Active
2.65 Å
Deactive
2.25 Å
Signal subtraction;
Focus-refinement
X
(see methods)
Focused 3D classification
T = 100;
Revert subtraction
Active-Q10
23,449 particles
(2.84 Å)
Active-apo
38,205 particles
(2.76 Å)
Deactive-ligand
235,957 particles
(2.30 Å)
Deactive-apo
23,583 particles
(2.81 Å)
Consensus
(2.08-4.69 Å)
2.30 Å
Signal subtraction;
3D refinement
Distal membrane domain
Proximal membrane domain
Hydrophilic domain
2.1
3.0
FSC
Resolution (1/Å)
(2.16-3.99 Å)
2.31 Å
(2.16-3.84 Å)
2.26 Å
(2.05-3.84 Å)
2.20 Å
Phenix
Combine focused maps
Deactive-ligand
Composite

Supplementary Fig. 3: Cryo-EM data processing – local classification. A flow chart of cryo-EM data processing from the active/deactive major classes to local substates (active-Q_{10}, active-apo, deactive-ligand, and deactive-apo) by local classification. As substantial heterogeneity was observed in the Q-binding sites of the active and deactive maps, the major classes were first signal subtracted to retain the hydrophilic arm, focus-refined, then 3D focus-classified without alignment using class-specific local masks. A tight mask generated from a tentative Q_{10} model was used for local classification for the active class, and a more generous mask from a provisional protein model (ND1, NDUFS2, and NDUFS7) encapsulating the Q-binding site region proved more successful for the deactive class. The consensus deactive-ligand map showed poor densities at the distal region of the membrane arm due to subtle differences in the 'openness' of the hydrophilic and membrane arms and stronger alignment to the former. For this reason, a composite map was made for the deactive-ligand class. The consensus map was split into three by signal subtraction and focus-refinement using the three overlaid masks shown in transparent colours. Local resolution maps are shown for the distal (left) and proximal (middle) membrane domains, and the peripheral domain (right). Local resolutions were estimated using the Local Resolution function in RELION and plotted using UCSF ChimeraX[7] with map thresholds set to 0.015. The coloured key on the right indicates the resolution in Å, and the map resolution ranges are indicated in brackets for the respective maps, in blue. RELION half-map (sky blue) FSC curves are shown for each domain. The RELION map sharpening *B*-factors (Å^2) for the consensus map, the distal and proximal membrane domains, and the peripheral domain were -58, -53, -49, and -51, respectively. The resulting composite map is shown in gray at a map threshold of 6.0. Composite maps were not made for the other classes either because there was little flexibility in the distal membrane domain (active-Q_{10} and active-apo) or because it did not lead to a marked improvement in map densities (deactive-apo and state 3). Where applicable, red boxes denote the final maps for the classes.

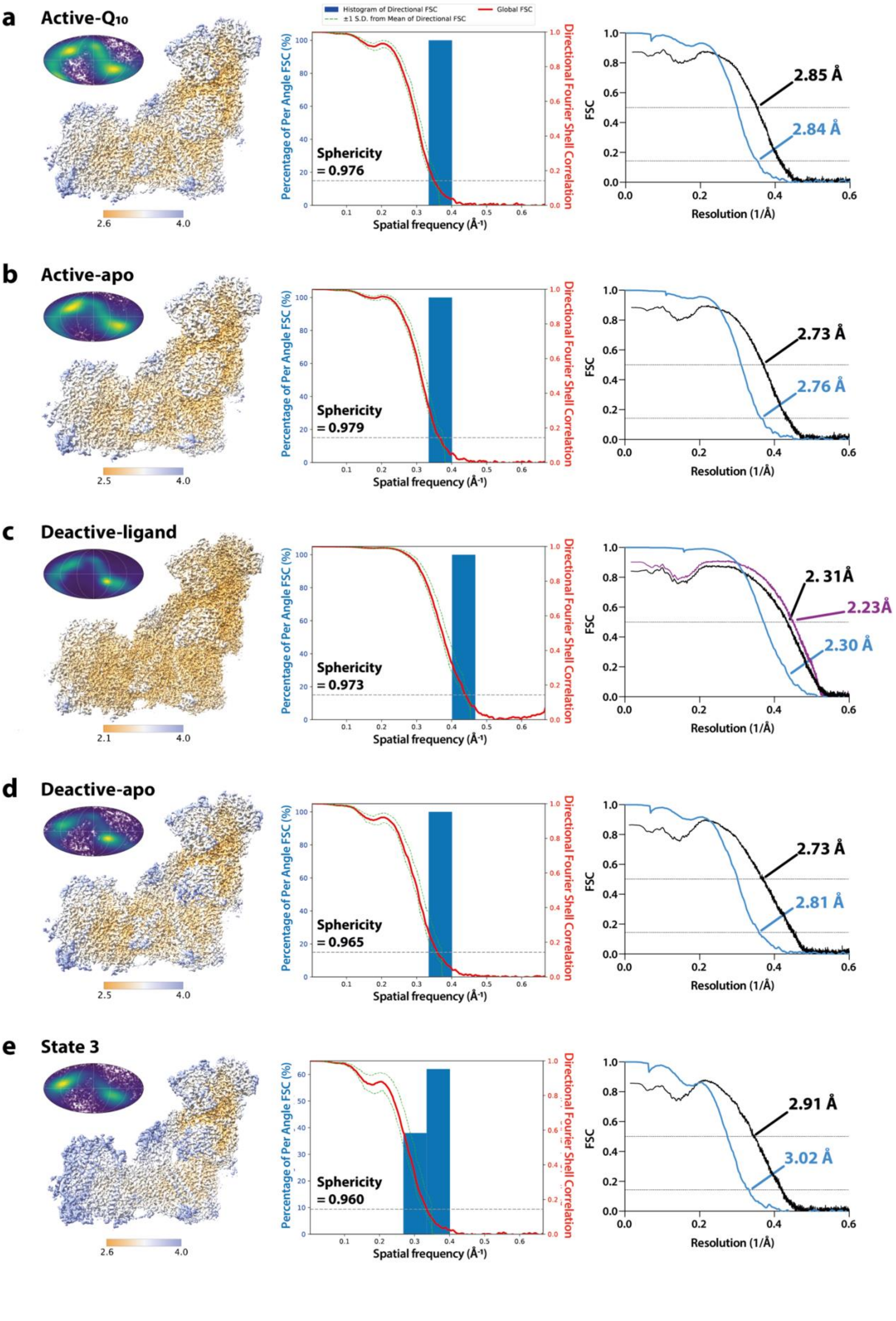
a
Active-Q10
2.6
4.0
Histogram of Directional FSC
±1 S.D. from Mean of Directional FSC
Global FSC
Sphericity
= 0.976
Percentage of Per Angle FSC (%)
Directional Fourier Shell Correlation
Spatial frequency (Å-1)
2.85 Å
2.84 Å
FSC
Resolution (1/Å)
b
Active-apo
2.5
4.0
Sphericity
= 0.979
2.73 Å
2.76 Å
c
Deactive-ligand
2.1
4.0
Sphericity
= 0.973
2. 31Å
2.23Å
2.30 Å
d
Deactive-apo
2.5
4.0
Sphericity
= 0.965
2.73 Å
2.81 Å
e
State 3
2.6
4.0
Sphericity
= 0.960
2.91 Å
3.02 Å

Supplementary Fig. 4: Local resolution maps, Mollweide projections, 3DFSC plots, and Fourier shell correlation curves for the five CxI-ND states. Local resolution consensus maps (left), Mollweide projections (left insert), histogram and directional FSC (3DFSC) plots (middle), and Fourier shell correlation (FSC) curves (right) are shown for the **(a)** active-Q_{10}, **(b)** active-apo, **(c)** deactive-ligand, **(d)** deactive-apo, and **(e)** state 3 structures. The deactive-ligand map is the composite map described in Supplementary Fig. 3. Local resolutions were estimated using the Local Resolution function in RELION and plotted using UCSF ChimeraX with map thresholds of 6.5, 6.0, 4.0, 6.5, and 6.5, respectively. Coloured keys indicate resolution in Å. Mollweide projections were plotted using Python and Matplotlib, and the degree of directional resolution anisotropy calculated using the 3DFSC program suite[9]. RELION half-map (sky blue) and model-map (black – locally-sharpened consensus maps; purple – focus-refined composite map) FSC curves are shown.

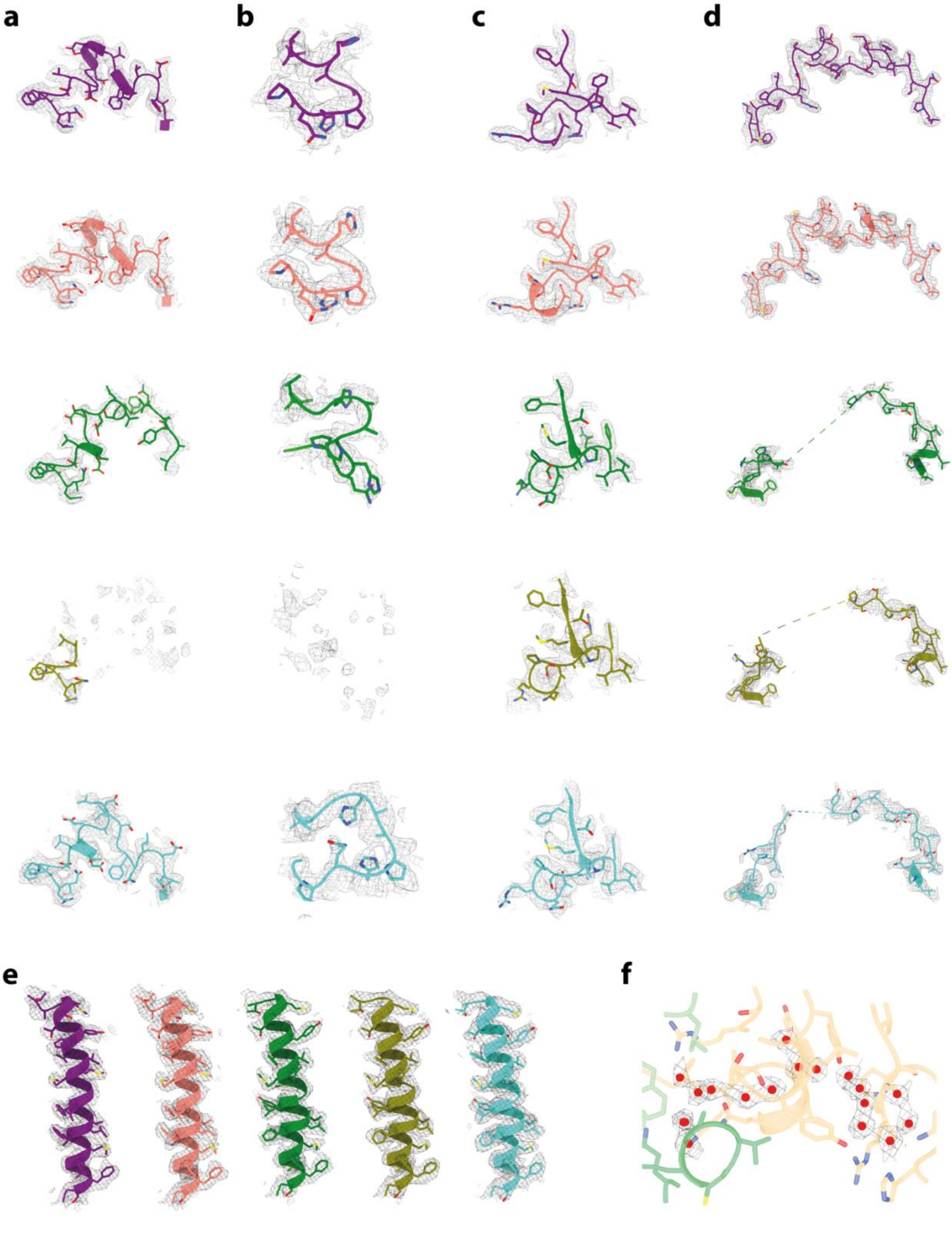

a
b
c
d
e
f

Supplementary Fig. 5: Densities in the CxI-ND maps for key elements that differ between the states and representative densities for water molecules. Densities for the **(a)** ND1-TMH5-6 loop (residues 194-217), **(b)** NDUFS2-β1-β2 loop (residues 52-62), **(c)** NDUFS7 residues 47-51 and 74-83, **(d)** ND3-TMH1-2 loop (residues 24-55), **(e)** ND6-TMH3 (residues 49-76), and **(f)** representative water molecules in the deactive-ligand state (at the interface between NDUFS2 (beige), NDUFS3 (pale green), and NDUFS8 (green)). All map densities are shown at map thresholds of 4.5 in UCSF ChimeraX[7]. Colours for the map densities are as follows: purple (active-Q_{10}), salmon (active-apo), green (deactive-ligand), olive (deactive-apo), and turquoise (state 3).

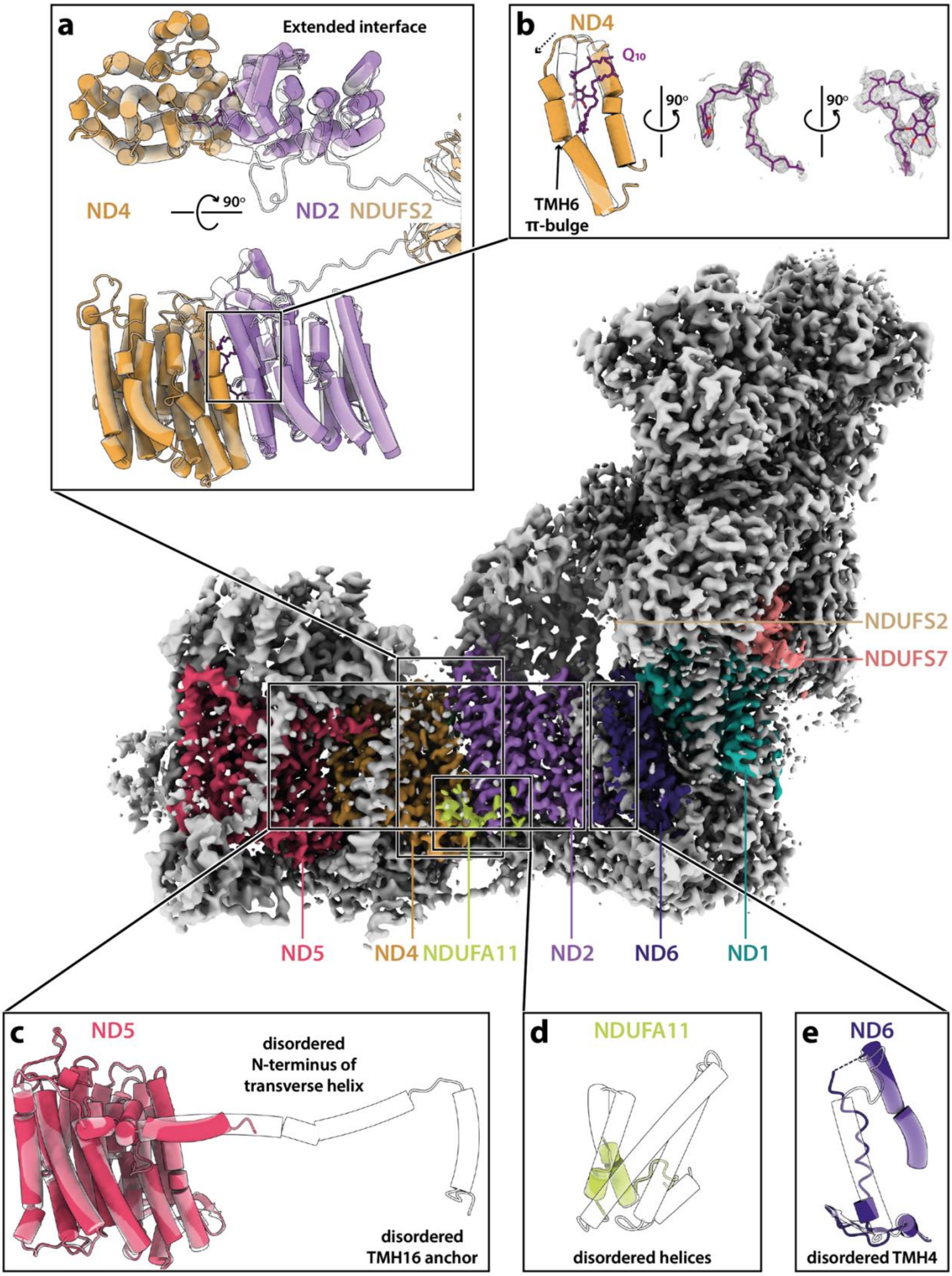
a
Extended interface
ND4
90°
ND2
NDUFS2
b
ND4
Q10
90°
90°
TMH6
π-bulge
NDUFS2
NDUFS7
ND5
ND4
NDUFA11
ND2
ND6
ND1
c
ND5
disordered
N-terminus of
transverse helix
disordered
TMH16 anchor
d
NDUFA11
disordered helices
e
ND6
disordered TMH4

Supplementary Fig. 6: Structural features of state 3 of bovine complex I. The cryo-EM density map for state 3 is presented at a map threshold of 6 in UCSF ChimeraX[7]. Cartoon and/or atomic representations are shown for **(a)** ND2, ND4, and NDUFS2, **(b)** ND4-TMH5-6 and Q_{10} (map threshold of 4.5), **(c)** ND5, **(d)** NDUFA11, and **(e)** ND6-TMH3-4. Models for state 3 are shown in solid colours and the deactive-ligand structure in transparent white with a black outline, aligned to the subunits shown. Conformational differences in the Q-binding site loops are shown in Fig. 4.

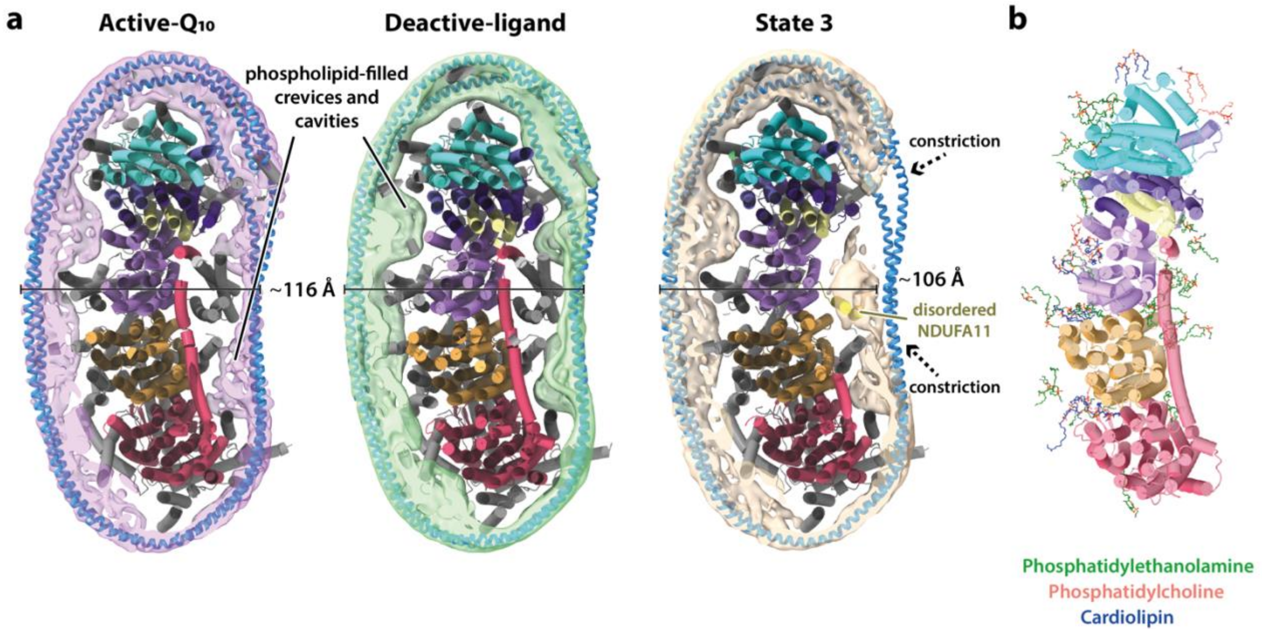

Supplementary Fig. 7: Structural features of complex I-reconstituted nanodiscs. a) Clipped top views of the active-Q_{10}, deactive-ligand and state 3 CxI-ND structures (cartoon) and their subtract-refined MSP2N2 nanodiscs, with the MSP2N2 polyalanine model overlaid. Potential regions in the state 3 structure where the MSP2N2 helices may 'wedge in' are indicated with dashed arrows. Lateral nanodisc widths are indicated. **b)** 42 phospholipids modelled in a representative CxI-ND structure (deactive-ligand), with the seven core membrane subunits represented as cartoons. All non-cardiolipin phospholipids were modelled as phosphatidylethanolamines unless density features indicated phosphatidylcholine to be more likely. All subunits are coloured as in Fig. 1.

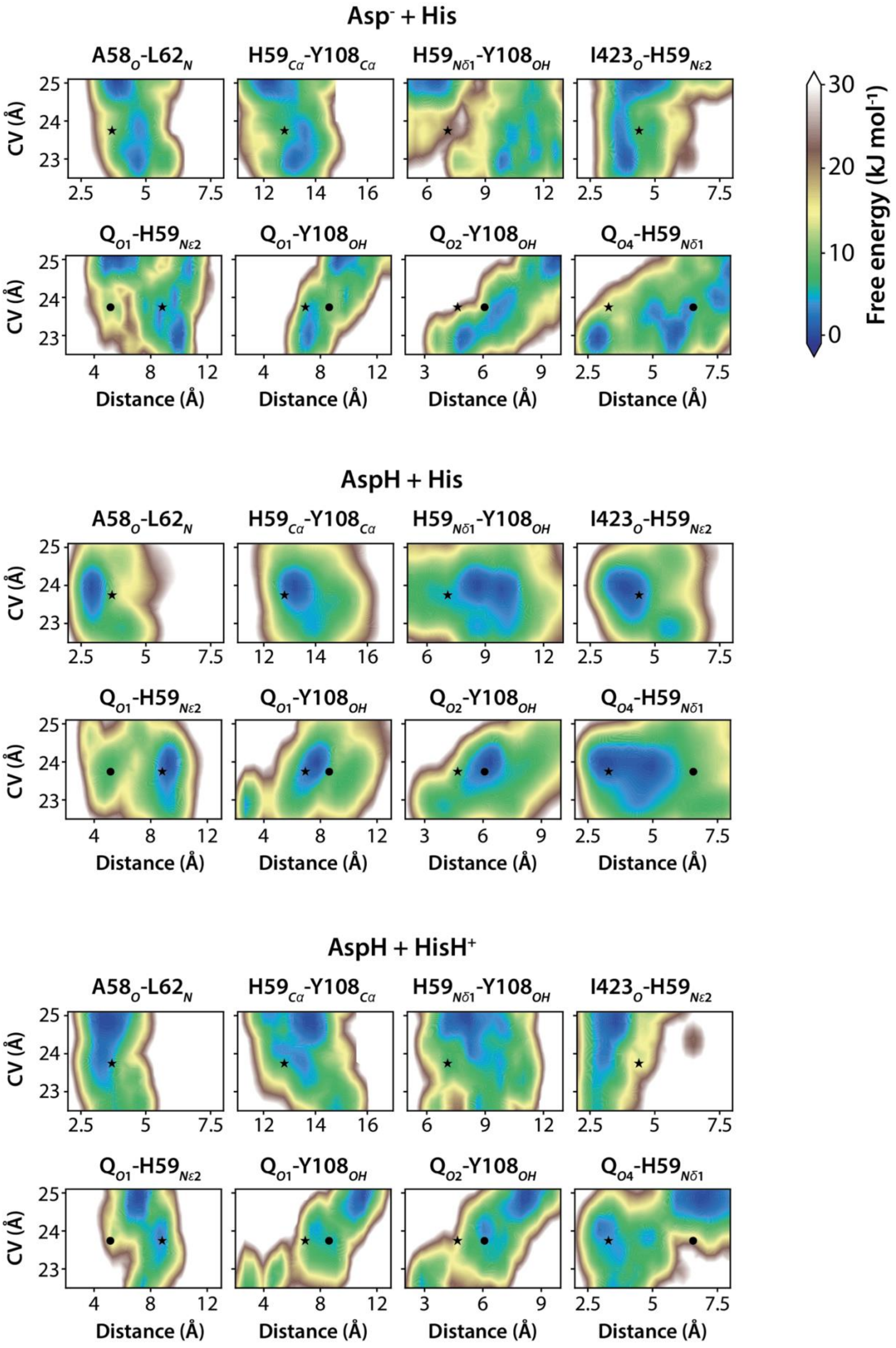

Asp- + His
AspH + His
AspH + HisH+
A58O-L62N
H59Cα-Y108Cα
H59Nδ1-Y108OH
I423O-H59Nε2
QO1-H59Nε2
QO1-Y108OH
QO2-Y108OH
QO4-H59Nδ1
CV (Å)
Distance (Å)
Free energy (kJ mol-1)

Supplementary Fig. 8. Free energy profiles for additional structural properties obtained from metadynamics simulations. Three combinations of sidechain protonation were simulated with Asp160^{NDUFS2} ionised (Asp^{-}) or protonated (AspH) and His59^{NDUFS2} neutral (His, $N_{\delta1}$-protonated π tautomer) or di-protonated (HisH^{+}, both $N_{\delta1}$- and $N_{\varepsilon2}$-protonated), as indicated above each pair of panel rows. CV describes the Q-headgroup position along the binding channel (Fig. 3b-d). Properties correspond to atom-pair distances shown on top of each column and symbols correspond to distances observed in active-Q_{10} cryo-EM models with primary (star) and flipped (bullet) Q-headgroup. The [AspH + His] charge-state displays free energy minima close (within 8 kJ mol^{-1}) to distances observed in active-Q_{10} cryo-EM models for all structural properties. This is not true for the other two charge-states for distances involving protein centres, for example distance His59^{NDUFS2}-$N_{\delta1}$–Tyr108^{NDUFS2}-OH for charge-state [Asp^{-} + His] and distance Ile423^{NDUFS2}-O–His59^{NDUFS2}-$N_{\varepsilon2}$ for charge-state [AspH + HisH^{+}], or involving the Q-headgroup, for example distances Q_{O2}–Tyr108^{NDUFS2}-OH and Q_{O4}–His59^{NDUFS2}-$N_{\delta1}$ for charge-state [Asp^{-} + His], or distances Q_{O1}–Tyr108^{NDUFS2}-OH and Q_{O2}–Tyr108^{NDUFS2}-OH for charge-state [AspH + HisH^{+}].

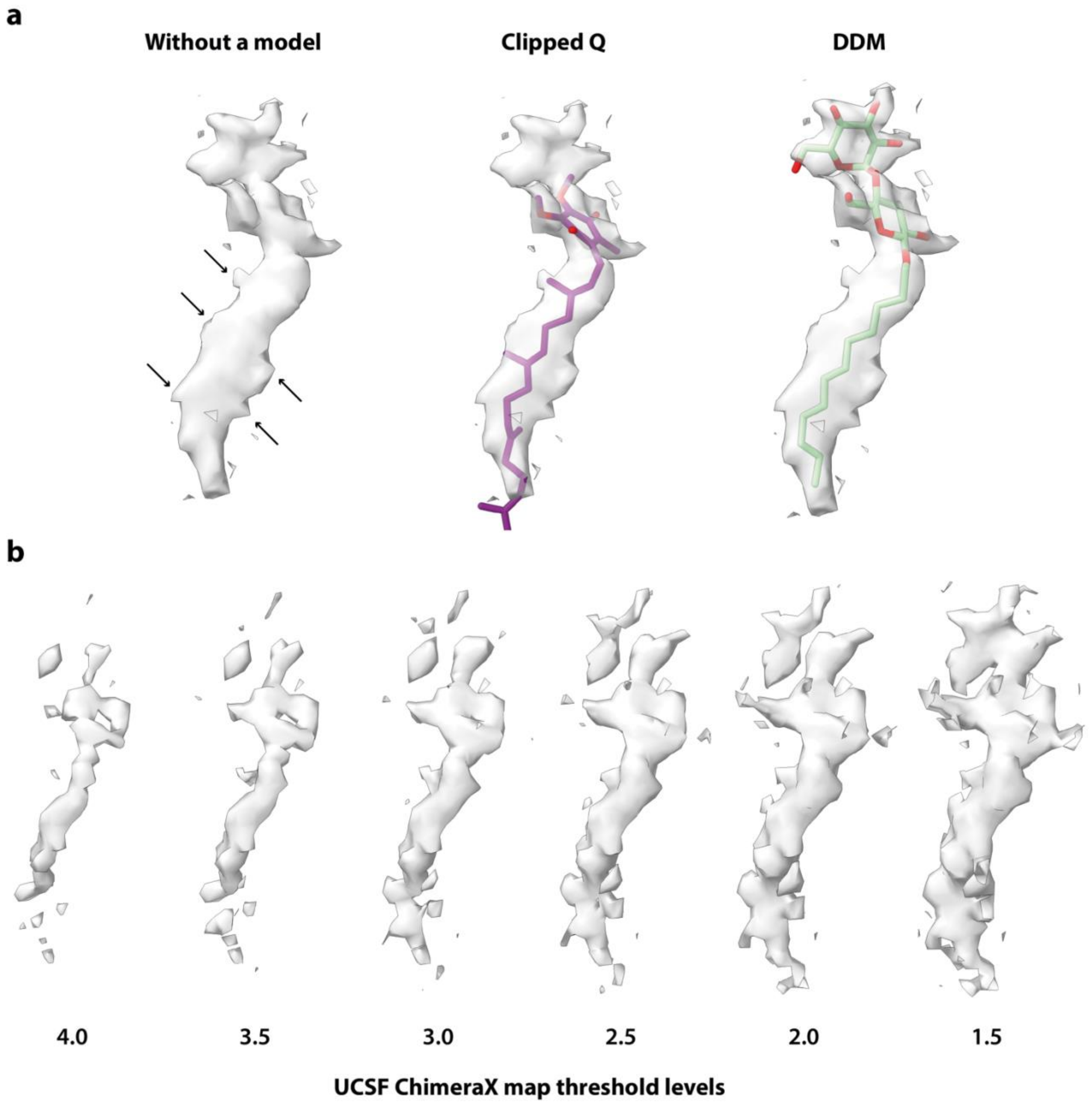

Supplementary Fig. 9: Features of the Coulomb potential density for the ligand in the deactive-ligand map. a) Focus-refined cryo-EM density of the ligand at a map threshold of 3 in an orthogonal view to Fig. 4c, showing zigzagged protrusions of the isoprenoid tail (indicated with arrows). Modelled Q (clipped) and DDM are shown. **b)** Locally-sharpened cryo-EM density of the ligand at decreasing map thresholds in UCSF ChimeraX[7], showing the weaker density for the second headgroup gradually emerging.

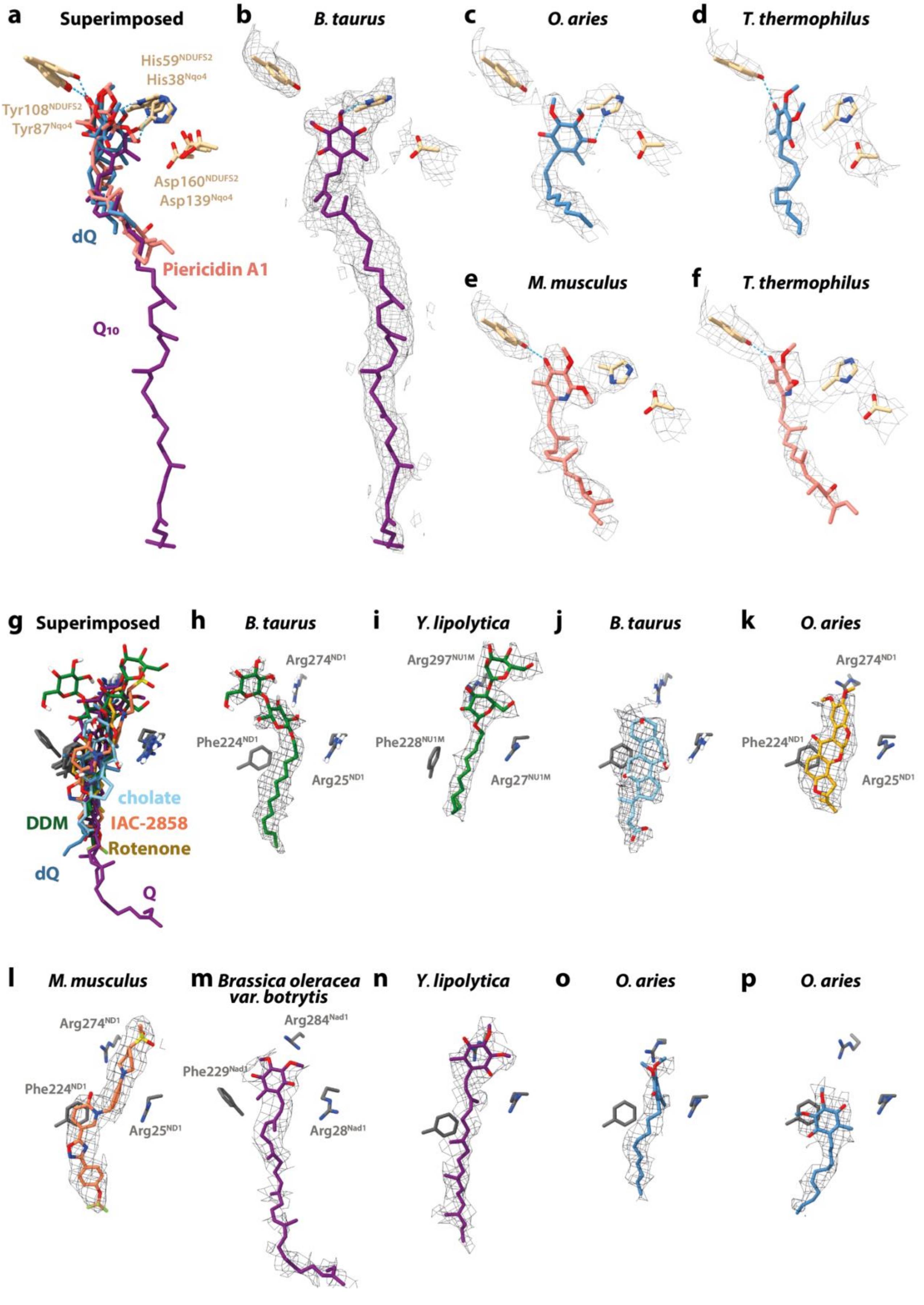

a Superimposed
b B. taurus
c O. aries
d T. thermophilus
His59NDUFS2
His38Nqo4
Tyr108NDUFS2
Tyr87Nqo4
Asp160NDUFS2
Asp139Nqo4
dQ
Piericidin A1
Q10
e M. musculus
f T. thermophilus
g Superimposed
h B. taurus
i Y. lipolytica
j B. taurus
k O. aries
Arg274ND1
Arg297NU1M
Arg274ND1
Phe224ND1
Phe228NU1M
Phe224ND1
Arg25ND1
Arg27NU1M
Arg25ND1
cholate
DDM
IAC-2858
Rotenone
dQ
Q
l M. musculus
m Brassica oleracea var. botrytis
n Y. lipolytica
o O. aries
p O. aries
Arg274ND1
Arg284Nad1
Phe224ND1
Phe229Nad1
Arg25ND1
Arg28Nad1

Supplementary Fig. 10: Comparisons between cryo-EM and X-ray crystal structures of ligands bound in the Q-binding site of complex I. a-f) Q_{10}, dQ, and piericidin A1 at the top of the Q-binding site. **a)** Superimposition of complex I-bound Q_{10}, dQ and piericidin A1 molecules shown alongside $Tyr108^{NDUFS2}$, $His59^{NDUFS2}$, and $Asp160^{NDUFS2}$ (mammalian numbering). **b)** Q_{10} in the bovine active-Q_{10} structure (cryo-EM map threshold 4.4 in UCSF ChimeraX[7]). **c)** dQ in ovine complex I in the 'closed' conformation reported to be undergoing turnover (PDB ID: 6ZKC) (cryo-EM map threshold 0.03, EMD-11244[6]). **d)** dQ in *T. thermophilus* complex I (PDB ID: 6I0D) (electron density map threshold 0.2[10]). **e)** Piericidin A1 in mouse complex I (PDB ID: 6ZTQ) (cryo-EM map threshold 0.015, EMD-11424[4]). **f)** Piericidin A1 in *T. thermophilus* complex I (PDB ID: 6Q8O) (electron density map threshold 0.06[10]). **g-p)** Various ligands bound at the entrance of the Q-binding site. **g)** Superimposition of complex I-bound DDM, cholate, rotenone, IACS-2858, $Q_{9\text{-}10}$, and dQ molecules shown alongside $Phe224^{ND1}$, $Arg274^{ND1}$, and $Arg25^{ND1}$ (mammalian numbering). **h)** DDM in the bovine deactive-ligand structure (cryo-EM map threshold 4). **i)** DDM in *Y. lipolytica* complex I (PDB ID: 6YJ4) (cryo-EM map threshold 0.025, EMD-10815[11]). **j)** Cholate in the bovine state 3 structure (cryo-EM map threshold 4.5). **k)** Rotenone in ovine complex I in the 'open' conformation (PDB ID: 6ZKM) (cryo-EM map threshold 0.05, EMD-11254[6]). **l)** IACS-2858 in mouse complex I (PDB ID: 7B93) (cryo-EM map threshold 0.04, EMD-12059[12]). **m)** Q_{10} in *Brassica oleracea var. botrytis* complex I (PDB ID: 7A23) (cryo-EM map threshold 0.009, EMD-11614[13]). **n)** Q_9 in *Y. lipolytica* complex I (PDB ID: 6RFR) (cryo-EM map threshold 0.01, EMD-4873[14]). **o)** dQ in ovine complex I in the 'closed' conformation reported to be undergoing turnover (PDB ID: 6ZKC) (cryo-EM map threshold 0.04, EMD-11244[6]). **p)** dQ in ovine complex I in the 'open' conformation reported to be undergoing turnover (PDB ID: 6ZKD) (cryo-EM map threshold 0.04, EMD-11245[6]).

Supplementary Note 1. Biochemical relevance of the state 3 structure.

Improvements in resolution of the third state of complex I, state 3, now enable a more comprehensive consideration of this poorly understood class of particles (Supplementary Fig. 5). Previously, for state 3 we described a loss of clear density for the C-terminal half of the ND5 transverse helix and its anchor helix (TMH16), much of the adjacent NDUFA11 subunit, and the N-terminal loop of NDUFS2[3,15]. The resolution of our early data was low (5.6 Å), but the same characteristics are conserved in our current higher resolution map (3.0 Å), so it is clear that these structural elements are disordered and/or have dissociated from the structured binding locations that they occupy on the complex in the active and deactive states. Our data now reveals also that ND6-TMH4 has lost its α-helical secondary structure in state 3, instead appearing as a poorly ordered loop, on the same exposed side of the complex as NDUFA11 and ND5-TMH16, extending the region affected by the disorder. Comparison of the nanodisc structures also shows clear differences in this region (Supplementary Fig. 6). Instead of clear densities stretching around NDUFA11 (as observed in the active and deactive states), the MSP2N2 helices in state 3 are not clearly resolved, consistent with substantial disorder in subunit NDUFA11 and/or with the MSP2N2 helices contracting inwards to occupy the space where NDUFA11 is usually observed. This change in nanodisc structure suggests that state 3 was present in the preparation before the enzyme was reconstituted into the nanodiscs. Therefore, it may result from destabilisation of the detergent-solubilised enzyme during its purification, but is not an artefact from cryo-EM grid preparation due to instability induced at the air-water interface[16–18]. Interestingly, NDUFA11 has been reported to be the only subunit in the membrane arm of complex I that undergoes rapid protein turnover in C2C12 myotubes[19], consistent with a higher propensity to dissociate *in vivo*. Furthermore, in the mammalian respirasome NDUFA11 is sandwiched in-between complex III and the core subunits of complex I[20–22], and the removal of complex III during solubilisation of the membrane may introduce additional instability to NDUFA11 and adjacent structures.

Comparing the three states of complex I resolved here shows clearly that state 3 has more in common with the deactive state than the active state. Globally, the apparent angle between the hydrophilic and hydrophobic domains of the complex is most acute in the active state, considerably more obtuse in the deactive state and similar, or greater still, in state 3. Key structural features that distinguish the deactive state from the active state are also present in state 3, including the π-bulge in ND6-TMH3, disordered ND3 TMH1-2 loop and structures around NDUFS7-Arg77. In contrast, the NDUFS2-β1-β2 and ND1 TMH5-6 loop conformations differ from those observed in both the active and deactive states. However, as features of the Q-binding site are known to be remodelled by ligands, it is unclear whether their conformations are a feature of state 3 or determined by ligand binding. Finally, the features discussed above that are disordered in state 3 are clearly ordered (and equivalent) in both the active and deactive states, and the ND2-ND4 interface is closed in both those states. Therefore, state 3 shares a substantial number of distinguishing features with the deactive state – but it shares none of them with the active state.

Due to the extra-relaxed and disordered characteristics of state 3, we propose to name this state 'slack' complex I in the future.

The three 'open' states described for native ovine complex I[6] exhibit all the key structural features observed in our deactive states, but also features that are specific to our state 3 structure. In particular, the state named open3 displays extensive disorder (evident as weak or absent densities) for NDUFA11, the C-terminal half of the ND5 transverse helix and its anchor helix (TMH16), the ~40 N-terminal residues of NDUFS2, and ND6-TMH4. These comparisons suggest that the open conformations of ovine complex I exist on a structural spectrum between our deactive state and state 3. Similar state 3-like characteristics are also observed in maps for ovine complex I reported to be in the deactive state, which we note was prepared by incubating a concentrated aliquot of the detergent-solubilised complex I at 32 °C for 30 min after anion exchange[6], rather than by deactivating the enzyme in its stabilising native membrane environment[2,5,23].

In state 3 the extended interface between subunits ND2 and ND4 contains an ordered Q_{10} molecule, accommodated by a π-bulge formed in ND4-TMH6. It is possible that the ND4-TMH6 π-bulge forms during catalysis, such that state 3 represents a relaxed state of a hitherto-uncharacterised intermediate, in which the π-bulge causes Tyr148^{ND4} to pivot away from its usual hydrogen bond to Glu559^{ND5}. By doing so it may destabilise the ND2-ND4 interface, propagating instability to the transverse helix and associated structures, resulting in the observed loss of structural integrity. The interfaces between NDUFA10, ND4, and ND2 have also been observed to accumulate strain in simulation work, and proposed to modulate conformational changes within the membrane domain[24]. Intriguingly, the position of the Q_{10}-headgroup bound at the ND2-ND4 interface in state 3 coincides with the position of a rotenone inhibitor molecule detected previously in ovine complex I open conformations[6]. Although it is difficult to conceive of any functional role for Q_{10} binding at this site, so distant from any of the enzyme's redox cofactors, it is possible that any molecule binding here could lock the position of Tyr148^{ND4} and thereby be inhibitory – if reorganisation of ND4-TMH6 really does occur on the catalytic cycle.

Loosening the structural constraints of the transverse helix in state 3 and opening up of the ND2-ND4 interface may alternatively allow the Q_{10}/rotenone molecule to enter in adventitiously. The propensity of the enzyme to fracture at this interface is highlighted by treatment of bovine complex I with the zwitterionic detergent *N,N*-dimethyldodecylamine *N*-oxide (LDAO) to form the ND4-ND5-containing subcomplex of the distal membrane domain known as subcomplex Iβ[25]. The same fragmentation occurred during crystallisation trials on the DDM-solubilised enzyme[26], yielding a structure of subcomplex Iβ[25] in which the C-terminal section of ND5 is highly disordered. The tendency of the ND4-ND5 module to dissociate was also illustrated by *Y. lipolytica* knock-out strain *nb8m*Δ (where NB8M is a homologue of subunit NDUFB7, adjacent to the C-terminus of ND5), in which the distal membrane domain was absent[27]. These observations argue that state 3 may represent an enzyme in the initial stages of degradation, not a catalytically relevant state.

To define the catalytic capability of state 3 it will be necessary to produce a homogeneous sample of it and characterise it both functionally and structurally. It is currently unclear how to achieve

this for bovine complex I, but we note that a structure we solved previously for complex I from rhesus macaque (*Macaca mulatta*)[15] was dominated by particles in this state (lacking clear densities for NDUFA11, the C-terminal half of the ND5 transverse helix and its anchor helix (TMH16), the N-terminus of NDUFS2, and parts of ND6-TMH4) – and the preparation exhibited only a very low activity of 0.5 µmol min^{-1} mg^{-1} [15]. Current evidence to suggest that state 3-like states do exist on the catalytic cycle is limited to their presence in a sample of ovine complex I treated with the substrates for turnover – however, similar states were present in the starting mixture and may simply not have responded to the addition of substrates[6]. Further investigations of complex I samples under established turnover conditions and in defined biochemical states will be necessary to finally establish the relevance of this intriguing enzyme state.

Supplementary Note 2: Minor differences between the structures of nanodisc-reconstituted and DDM-solubilised complex I

First, in all CxI-ND maps, the tip of the β-hairpin in the ND6 TMH4-5 loop on the intermembrane space face is shifted by 3-3.5 Å towards subunit ND2, so that it makes contact with the ND2-TMH4-5 helix-loop-helix motif and ND4L-TMH1. In the DDM-solubilised reference active-state structure[1], there is an unmodelled density between the two structural elements that resembles a DDM molecule displacing the ND6-β-hairpin. The β-hairpin motif is not present in *M. musculus* complex I[5,23], while in the lauryl maltose neopentyl glycol (LMNG)-solubilised ovine enzyme[6] its conformation matches the CxI-ND conformation.

Second, a density for a steroid molecule (modelled as a cholate, which was added at the reconstitution step) is present in all CxI-ND maps at the interface between subunits ND5 and NDUFB6. The nearby NDUFB6 matrix helix/loop is resolved for the first time here, perhaps as a result of the structure being stabilised by incorporation of the additional cholate molecule and enclosed by the adjacent MSP2N2 helices – although the previously unstructured region makes remarkably few direct contacts with any other subunits.

Third, the C-terminal peptide of mammalian subunit NDUFS1 (ca. 10 residues) is also resolved for the first time here, with the terminal $Cys704^{NDUFS1}$ residue lodged between subunits NDUFS1, NDUFS6, and NDUFS8. The absence of DDM in the sample buffer may be responsible for the improved resolution of this peptide in the nanodisc structures, on the outside of the hydrophilic domain.

Supplementary References

1. Bridges, H. R., Blaza, J. N., Yin, Z., Chung, I. & Hirst, J. Bovine complex I in the active state at 3.1 Å. (2022) DOI: 10.2210/pdb7QSD/pdb.
2. Blaza, J. N., Vinothkumar, K. R. & Hirst, J. Structure of the Deactive State of Mammalian Respiratory Complex I. *Structure* **26**, 312–319 (2018).
3. Zhu, J., Vinothkumar, K. R. & Hirst, J. Structure of mammalian respiratory complex I. *Nature* **536**, 354–358 (2016).
4. Bridges, H. R. *et al.* Structure of inhibitor-bound mammalian complex I. *Nat. Commun.* **11**, 5261 (2020).
5. Yin, Z. *et al.* Structural basis for a complex I mutation that blocks pathological ROS production. *Nat. Commun.* **12**, 707 (2021).
6. Kampjut, D. & Sazanov, L. A. The coupling mechanism of mammalian respiratory complex I. *Science* **370**, eabc4209 (2020).
7. Goddard, T. D. *et al.* UCSF ChimeraX: Meeting modern challenges in visualization and analysis. *Protein Sci.* **27**, 14–25 (2018).
8. Schrodinger LLC. The PyMOL Molecular Graphics System, Version 2.4.1. (2021).
9. Tan, Y. Z. *et al.* Addressing preferred specimen orientation in single-particle cryo-EM through tilting. *Nat. Methods* **14**, 793–796 (2017).
10. Gutiérrez-Fernández, J. *et al.* Key role of quinone in the mechanism of respiratory complex I. *Nat. Commun.* **11**, 4135 (2020).
11. Grba, D. N. & Hirst, J. Mitochondrial complex I structure reveals ordered water molecules for catalysis and proton translocation. *Nat. Struct. Mol. Biol.* **27**, 892–900 (2020).
12. Chung, I. *et al.* Cork-in-bottle mechanism of inhibitor binding to mammalian complex I. *Sci. Adv.* **7**, eabg4000 (2021).
13. Soufari, H., Parrot, C., Kuhn, L., Waltz, F. & Hashem, Y. Specific features and assembly of the plant mitochondrial complex I revealed by cryo-EM. *Nat. Commun.* **11**, 5195 (2020).
14. Parey, K. *et al.* High-resolution cryo-EM structures of respiratory complex I: Mechanism, assembly, and disease. *Sci. Adv.* **5**, eaax9484 (2019).
15. Agip, A.-N. A., Blaza, J. N., Fedor, J. G. & Hirst, J. Mammalian Respiratory Complex I Through the Lens of Cryo-EM. *Annu. Rev. Biophys.* **48**, 165–184 (2019).
16. Glaeser, R. M. & Han, B.-G. Opinion: hazards faced by macromolecules when confined to thin aqueous films. *Biophys. Reports* **3**, 1–7 (2017).
17. Noble, A. J. *et al.* Routine single particle CryoEM sample and grid characterization by tomography. *Elife* **7**, 1–42 (2018).
18. D'Imprima, E. *et al.* Protein denaturation at the air-water interface and how to prevent it. *Elife* **8**, 1–18 (2019).
19. Krishna, S. *et al.* Identification of long-lived proteins in the mitochondria reveals increased stability of the electron transport chain. *Dev. Cell* **56**, 2952–2965 (2021).
20. Gu, J. *et al.* The architecture of the mammalian respirasome. *Nature* **537**, 639–643 (2016).

21. Wu, M., Gu, J., Guo, R., Huang, Y. & Yang, M. Structure of Mammalian Respiratory Supercomplex $I_1III_2IV_1$. *Cell* **167**, 1598–1609 (2016).

22. Letts, J. A., Fiedorczuk, K., Degliesposti, G., Skehel, M. & Sazanov, L. A. Structures of Respiratory Supercomplex $I+III_2$ Reveal Functional and Conformational Crosstalk. *Mol. Cell* **75**, 1131–1146 (2019).

23. Agip, A.-N. A. *et al.* Cryo-EM structures of complex I from mouse heart mitochondria in two biochemically defined states. *Nat. Struct. Mol. Biol.* **25**, 548–556 (2018).

24. Di Luca, A. & Kaila, V. R. I. Molecular strain in the active/deactive-transition modulates domain coupling in respiratory complex I. *Biochim. Biophys. Acta - Bioenerg.* **1862**, 148382 (2021).

25. Sazanov, L. A., Peak-Chew, S. Y., Fearnley, I. M. & Walker, J. E. Resolution of the Membrane Domain of Bovine Complex I into Subcomplexes: Implications for the Structural Organization of the Enzyme. *Biochemistry* **39**, 7229–7235 (2000).

26. Zhu, J. *et al.* Structure of subcomplex Iβ of mammalian respiratory complex I leads to new supernumerary subunit assignments. *Proc. Natl. Acad. Sci.* **112**, 12087–12092 (2015).

27. Dröse, S. *et al.* Functional Dissection of the Proton Pumping Modules of Mitochondrial Complex I. *PLoS Biol.* **9**, e1001128 (2011).
