# Supplementary material for: Cryo-EM structures define ubiquinone-10 binding to mitochondrial complex I and conformational transitions accompanying Q-site occupancy
Source: Nat Commun. 2022 May 19;13:2758. doi: 10.1038/s41467-022-30506-1 (PMC9120487; doi:10.1038/s41467-022-30506-1)
Supplement: Supplementary file 3 — Description of Additional Supplementary Files [file 41467_2022_30506_MOESM3_ESM.pdf]

Description of Additional Supplementary Files

File Name: Supplementary Data 1
Description: **Milestone configurations used to calculate the collective variable (CV).** Co-ordinates of heavy atoms in the Q-headgroup and the Cα atoms of residues in subunits NDUFS7, NDUFS2 and ND1 exposed to the Q-binding channel.
