# Supplementary material for: Cryo-EM structures define ubiquinone-10 binding to mitochondrial complex I and conformational transitions accompanying Q-site occupancy
Source: Nat Commun. 2022 May 19;13:2758. doi: 10.1038/s41467-022-30506-1 (PMC9120487; doi:10.1038/s41467-022-30506-1)
Supplement: Supplementary file 5 — Reporting Summary [file 41467_2022_30506_MOESM5_ESM.pdf]

nature portfolio

Corresponding author(s): Hirst, J

Last updated by author(s): Apr 29, 2022

Reporting Summary

Nature Portfolio wishes to improve the reproducibility of the work that we publish. This form provides structure for consistency and transparency in reporting. For further information on Nature Portfolio policies, see our Editorial Policies and the Editorial Policy Checklist.

Statistics

For all statistical analyses, confirm that the following items are present in the figure legend, table legend, main text, or Methods section.

n/a	Confirmed	
☐	☒	The exact sample size (*n*) for each experimental group/condition, given as a discrete number and unit of measurement
☐	☒	A statement on whether measurements were taken from distinct samples or whether the same sample was measured repeatedly
☒	☐	The statistical test(s) used AND whether they are one- or two-sided<br>*Only common tests should be described solely by name; describe more complex techniques in the Methods section.*
☒	☐	A description of all covariates tested
☒	☐	A description of any assumptions or corrections, such as tests of normality and adjustment for multiple comparisons
☐	☒	A full description of the statistical parameters including central tendency (e.g. means) or other basic estimates (e.g. regression coefficient) AND variation (e.g. standard deviation) or associated estimates of uncertainty (e.g. confidence intervals)
☒	☐	For null hypothesis testing, the test statistic (e.g. *F*, *t*, *r*) with confidence intervals, effect sizes, degrees of freedom and *P* value noted<br>*Give P values as exact values whenever suitable.*
☒	☐	For Bayesian analysis, information on the choice of priors and Markov chain Monte Carlo settings
☒	☐	For hierarchical and complex designs, identification of the appropriate level for tests and full reporting of outcomes
☒	☐	Estimates of effect sizes (e.g. Cohen's *d*, Pearson's *r*), indicating how they were calculated

Our web collection on statistics for biologists contains articles on many of the points above.

Software and code

Policy information about availability of computer code

Data collection	EPU 2.7, Softmax Pro
Data analysis	RELION 3.1, CTFFIND-4.1, Python, Matplotlib, Phenix 1.18.2-3874 and 1.19-4092, Coot 0.9.4-pre and 0.9.4.2-pre, Curlew, ISOLDE 1.2.2, EMRinger, MolProbity 4.4, PyMol 2.5.2, CASTp, UCSF ChimeraX 1.3.0, MapQ, PropKa, GROMACS 2020.3, PLUMED 2.6.1.

For manuscripts utilizing custom algorithms or software that are central to the research but not yet described in published literature, software must be made available to editors and reviewers. We strongly encourage code deposition in a community repository (e.g. GitHub). See the Nature Portfolio guidelines for submitting code & software for further information.

Data

Policy information about availability of data

All manuscripts must include a data availability statement. This statement should provide the following information, where applicable:

- Accession codes, unique identifiers, or web links for publicly available datasets
- A description of any restrictions on data availability
- For clinical datasets or third party data, please ensure that the statement adheres to our policy

The data generated in this study have been deposited in the EMDB and PDB databases under the following accession codes: EMD-14132 and PDB ID: 7QSK (active-Q10), EMD-14133 and PDB ID: 7QSL (active-apo), EMD-14134 and PDB ID: 7QSM (deactive-ligand; composite), EMD-14135, EMD-14136, EMD-14137, and EMD-14138 (deactive-ligand; consensus, hydrophilic domain, proximal and distal membrane domains, respectively), EMD-14139 and PDB ID: 7QSN (deactive-apo), and EMD-14140 and PDB ID: 7QSO (state 3). Related data accession codes: EMD-14127 and PDB:7QSD (DDM-solubilised complex I).

Field-specific reporting

Please select the one below that is the best fit for your research. If you are not sure, read the appropriate sections before making your selection.

[x] Life sciences [ ] Behavioural & social sciences [ ] Ecological, evolutionary & environmental sciences

For a reference copy of the document with all sections, see nature.com/documents/nr-reporting-summary-flat.pdf

Life sciences study design

All studies must disclose on these points even when the disclosure is negative.

Sample size	Sample size was not predetermined. For cryo-EM structure determination, sample sizes were those required for the target resolution. Cryo-EM data were collected from two datasets originating from a single protein purification. Entire datasets contained 804,367 and 382,037 particles picked, which were then filtered by 2D and 3D classification. Final classes contained a total 343,213 particles.
Data exclusions	After particle classification, data for non-protein or damaged protein images were removed (see Methods). No data were excluded from biochemical or computational experiments.
Replication	Cryo-EM data was measured from two datasets. During 3D refinement two randomly divided half datasets were processed independently, and combined to give rise to the final structures. The final resolution of the structure is assessed by comparing the two independent half maps. Kinetic measurements were of two to four technical replicates. All attempts to replicate experiments were successful.
Randomization	Randomisation is not relevant to the majority of experiments in this study as samples were not grouped for analysis. Randomisation was used for cryo-EM, where two randomly divided half datasets were generated during 3D refinement, as a standard approach implemented in RELION.
Blinding	Not applicable as samples were not grouped for analysis.

Reporting for specific materials, systems and methods

We require information from authors about some types of materials, experimental systems and methods used in many studies. Here, indicate whether each material, system or method listed is relevant to your study. If you are not sure if a list item applies to your research, read the appropriate section before selecting a response.

Materials & experimental systems

n/a	Involved in the study
[x]	[ ] Antibodies
[x]	[ ] Eukaryotic cell lines
[x]	[ ] Palaeontology and archaeology
[x]	[ ] Animals and other organisms
[x]	[ ] Human research participants
[x]	[ ] Clinical data
[x]	[ ] Dual use research of concern

Methods

n/a	Involved in the study
[x]	[ ] ChIP-seq
[x]	[ ] Flow cytometry
[x]	[ ] MRI-based neuroimaging
